# Geometry function of a linear brachytherapy source

**DOI:** 10.1120/jacmp.v2i2.2615

**Published:** 2001-03-01

**Authors:** R. Paul King, R. Scott Anderson, Michael D. Mills

**Affiliations:** ^1^ Jeff Anderson Regional Medical Center 1724 23rd Avenue, Bldg. C Meridian Mississippi 39301; ^2^ James Graham Brown Cancer Center 529 South Jackson Street Louisville Kentucky 40202

**Keywords:** brachytherapy, geometry function, palladium, TG43

## Abstract

An equation is derived for the TG43 geometry function, *G(r,θ)*, of a linear brachytherapy source in terms of its active length. This equation is validated by comparison to published values. It is then used to calculate values of the geometry function for the Model $200{\;}^{103}\text{Pd}$ seed, which is a segmented linear source.

PACS number(s): 87.53.–j

## I. DERIVATION

The TG43 formalism^1^ expresses the dose rate distribution around a cylindrically symmetric brachytherapy source as separable into component functions.

(1) $$\dot{D}(r,\theta )=S_{k}\Lambda \left[\frac{G(r,\theta )}{G(r_{0},\theta_{0})}\right]g(r)F(r,\theta ).$$

$S_{k}$ is the air kerma strength, Λ is the dose rate constant, *G(r,θ)* is the geometry function, and *G(r,θ)* is the anisotropy function.

The geometry function is normalized to a point 1 cm from the source center along a perpendicular bisector. Its purpose is to quantify the effect of the spatial arrangement of radioactive material on the dose distribution. If the source extent is assumed to be negligible, then the geometry function is modeled by the inverse square relationship. For a finite line source, the geometry function is calculated as follows:

(2) $$G(r,\theta )=\frac{\beta }{Lr\sin(\theta )}.$$

β is the angle subtended by the point and the source endpoints and *L* is the active source length.

The report of TG43 provides a tabulation of the geometry function for a 3‐mm line source and leaves it for the reader to calculate this function for other linear sources. The activity distribution of the Model $200{\;}^{103}\text{Pd}$ seed (Theragenics Corporation, Norcross, GA) can be described as a 2.8 mm segmented line source with a 1.0 mm inactive region centered between two active regions of 0.9 mm length^2^ The geometry function of this source is not readily calculated from Eq. (2). The objective of the present work is to recast Eq. (2) into a function of *r, θ,* and *L* and calculate the geometry function of the Model $200{\;}^{103}\text{Pd}$ seed. Recasting Eq. (2) requires geometric analysis of the source configuration.

**Figure 1:**
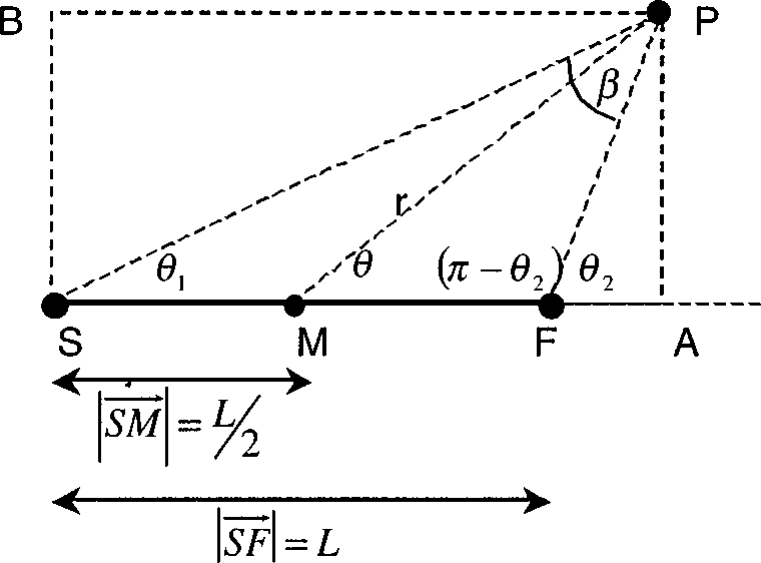
Source geometry.

Consider a linear source whose end points are labeled *S* and *F* as shown in Fig. 1. The midpoint, *M*, is located midway between *S* and *F* and serves as the origin of the coordinate system. Point *P* is located in a position which is arbitrary with respect to the source. Using the conventions of TG43, line segments connecting *S, M,* and *F* with *P* form angles of $\theta_{1},\theta ,\theta_{2}$, with the long axis of the source. The magnitude of the angle ∠ *SPF* is designated as β.

If the line segment connecting *M* and *P* has length *r*, then the position of *P* can be referred to as *P(r,θ)*. The relationship between $\beta ,\theta_{1}$, and $\theta_{2}$ can be found by recognizing that the sum of the angles in the triangle ΔASFP is it.

(3) $$\theta_{1}+\beta +(\pi -\theta_{2})=\pi ∴\beta =\theta_{2}-\theta_{1}.$$

If we treat the points *S* and *P* as diametrically opposed corners of a rectangle, this provides the means to eliminate $\theta_{1}$ and $\theta_{2}$ from the expression for β. The value of $\theta_{2}$ is expressed in terms of *r* and θ using the trigonometric definitions,

(4) $$|\vec{AP}|=r*\sin(\theta ),$$

(5) $$|\vec{MA}|=r*\cos(\theta ).$$

Thus

(6) $$|\vec{BP}|=|\vec{SA}|=(|\vec{SM}|+|\vec{MA}|)=[r*\cos(\theta )+L/2],$$

(7) $$|\vec{FA}|=(|\vec{BP}|-L)=[r*\cos(\theta )+L/2-L]=[r*\cos(\theta )-L/2],$$

(8) $$\theta_{2}=\tan^{-1}\left(\frac{\left|\vec{AP}\right|}{\left|\vec{FA}\right|}\right)=\tan^{-1}\left(\frac{r*\sin(\theta )}{r*\cos(\theta )-L/2}\right).$$

The law of sines is used to express β n terms of the independent variables

(9) $$\frac{\left|\vec{SP}\right|}{\sin(\pi -\theta_{2})}=\frac{L}{\sin(\beta )}.$$

From the property of the sine function

(10) $$\sin(\pi -\theta_{2})=\sin(\theta_{2}),$$

(11) $$\sin(\beta )=\frac{L*\sin(\theta_{2})}{\left|\vec{SP}\right|}.$$

Since the points *S, A*, and *P* form a right triangle, the pythagorean theorem is used to calculate the distance from *S* to *P*.

(12) $$|\vec{SP}|=\sqrt{{\left[r*\sin(\theta )\right]}^{2}+{\left[r*\cos(\theta )+L/2\right]}^{2}}$$

Substituting Eq. (8) into Eq. (11),

(13) $$\beta =\sin^{-1}\left(\frac{L*\sin(\tan^{-1}\{[r*\sin(\theta )]/[r*\cos(\theta )-L/2]\})}{\sqrt{{\left[r*\sin(\theta )\right]}^{2}+{\left[r*\cos(\theta )+L/2\right]}^{2}}}\right).$$

Since β is given in terms of *r*, *θ*, and the constant *L; G(r, θ)* can also be expressed in terms of *r*, *θ*, and *L*.

(14) $$G(r,\theta )=\frac{\sin^{-1}\left(\frac{L*\sin(\tan^{-1}\{[r*\sin(\theta )]/[r*\cos(\theta )-L/2]\})}{\sqrt{{\left[r*\sin(\theta )\right]}^{2}+{\left[r*\cos(\theta )+L/2\right]}^{2}}}\right)}{L*r*\sin(\theta )}.$$

Thus to tabulate the geometry function of a line source in terms of the polar coordinates, *r* and 4tH, we need only know the length of the source.

## II. VALIDATION

The geometry function for the 3‐mm line source presented in TG43's Table V was used as a benchmark against which to validate Eq. (14). The line source geometry function presented in TG43 is presented as multiplied by $r^{2}$. In this way, the tabulated value demonstrates the deviation of the behavior of the small line source from that of a true point source. It also presents a slowly varying function for which linear interpolation can be used to determine values intermediate to those presented in the table. It is noteworthy that in the general formalism for dose calculation presented in TG43's Eq. (2), the geometry function is used only as normalized to its value at the reference point. Thus this normalization is present in Table I such that the values presented are $\left[r^{2}*G(r,\theta )/(r_{0},\theta_{0})\right]$. For a true point source, each of the values in such a table would be unity.

**Table I:** Normalized geometry function for a 3‐mm line source $\left[r^{2}*G(r,\theta )/(r_{0},\theta_{0})\right]$.

	r=0.5 cm	1 cm	2 cm	3 cm	4 cm	5 cm	6 cm	7 cm	8 cm	9 cm
θ=0°	1.1071	1.0306	1.0132	1.0100	1.0089	1.0084	1.0081	1.0079	1.0078	1.0077
10°	1.1023	1.0297	1.0129	1.0099	1.0088	1.0083	1.0081	1.0079	1.0078	1.0077
20°	1.0889	1.0269	1.0123	1.0096	1.0087	1.0082	1.0080	1.0078	1.0078	1.0077
30°	1.0694	1.0227	1.0112	1.0091	1.0084	1.0081	1.0079	1.0078	1.0077	1.0076
40°	1.0471	1.0176	1.0100	1.0086	1.0081	1.0079	1.0077	1.0077	1.0076	1.0076
50°	1.0252	1.0123	1.0087	1.0080	1.0078	1.0077	1.0076	1.0076	1.0075	1.0075
60°	1.0059	1.0074	1.0074	1.0075	1.0075	1.0075	1.0075	1.0075	1.0075	1.0075
70°	0.9912	1.0034	1.0064	1.0070	1.0072	1.0073	1.0073	1.0074	1.0074	1.0074
80°	0.9819	1.0009	1.0058	1.0067	1.0070	1.0072	1.0073	1.0073	1.0074	1.0074
90°	0.9788	1.0000	1.0056	1.0066	1.0070	1.0072	1.0072	1.0073	1.0073	1.0074

Note that Eq. (14) has a singularity, i.e., a point where its value is indeterminate [0/0], when the angle is zero. The values at this angle were determined by numerically evaluating the limit as the angle approached zero. In other words, a value was used to approximate zero, which was sufficiently small that the value of the geometry function (to the fourth decimal place) did not change when the angle was doubled or halved.

These values are found to be in agreement with the corresponding entries in TG43's Table V to within 0.1% at all points except (r=0.5,θ=60°) where the discrepancy is 8.3%. At this point, the result of the present work is in agreement with the correction to TG43 provided by Rivard.^3^ Equation (14) is seen to predict results consistent with values known to be accurate.

## III. APPLICATION

To calculate the geometry function of the Model $200{\;}^{103}\text{Pd}$ seed using Eq. (14), the calculation takes three steps. First, the geometry function is calculated for the 2.8 mm outer length and the result is scaled by the outer length. Second, the geometry function is calculated for the 1.0 mm inner length and the result is scaled by the inner length. Finally, the scaled results are subtracted and normalized to the reference point. The resulting values are presented in Table II.

**Table II:** Normalized geometry function for the Model $200{\;}^{103}\text{Pd}$ Source $\left[r^{2}*G(r,\theta )/G(r_{0},\theta_{0})\right]$.

	r=0.5 cm	1 cm	2 cm	3 cm	4 cm	5 cm	6 cm	7 cm
θ=0°	1.1376	1.0397	1.0171	1.0129	1.0115	1.0109	1.0105	1.0103
10°	1.1316	1.0384	1.0168	1.0128	1.0114	1.0108	1.0105	1.0103
20°	1.1146	1.0348	1.0159	1.0124	1.0112	1.0107	1.0104	1.0102
30°	1.0898	1.0294	1.0146	1.0119	1.0109	1.0105	1.0102	1.0101
40°	1.0613	1.0228	1.0130	1.0111	1.0105	1.0102	1.0100	1.0099
50°	1.0329	1.0159	1.0113	1.0104	1.0101	1.0099	1.0099	1.0098
60°	1.0079	1.0096	1.0097	1.0097	1.0097	1.0097	1.0097	1.0097
70°	0.9886	1.0044	1.0084	1.0091	1.0093	1.0095	1.0095	1.0096
80°	0.9764	1.0011	1.0075	1.0087	1.0091	1.0093	1.0094	1.0095
90°	0.9723	1.0000	1.0072	1.0086	1.0091	1.0093	1.0094	1.0095

Before subtraction, the component geometry functions are scaled by segment length, to reflect a uniform linear activity density. Why these segments should be scaled by length can be understood by recognizing that the normalized geometry function is a multiplicative term in the calculation of dose. For an idealized source with a uniform linear activity density of unity, a unit dose rate constant, no self‐attenuation, and a distance‐independent radial dose function, the dose would be calculated as the normalized geometry function multiplied by length. Since the superposition principle applies to the quantity absorbed dose, the dose delivered by individual source components are added and subtracted as those components are introduced and removed. Since, for a source of uniform linear activity density, the length scales the activity; it also scales the dose and thus the net‐effective normalized geometry function.

The normalized geometry function is one component of the TG43 formalism used to estimate the dose at points surrounding a Model $200{\;}^{103}\text{Pd}$ source. Improvements in the accuracy of the geometry function translate into improvements in the estimation of dose. The magnitude of the improvement is position dependent. Improvements in $\left[r^{2}*G(r,\theta )/G(r_{0},\theta_{0})\right]$ of up to 3% are seen as compared to treating this segmented source as a 3 mm uniform line source. Up to 14% are seen when compared to use of a point source approximation.
